# Vortioxetine in children and adolescents with major depressive disorder: 6-month and 18-month open-label, flexible-dose, long-term extension studies

**DOI:** 10.1007/s00787-024-02560-1

**Published:** 2024-09-06

**Authors:** Melissa P. DelBello, Robert L. Findling, Michael Huss, Oscar Necking, Maria L. Petersen, Simon N. Schmidt, Monika Rosen

**Affiliations:** 1 Department of Psychiatry and Behavioral Neuroscience, University of Cincinnati College of Medicine, 260 Stetson Ave. Suite 3200, Cincinnati, Ohio 45219 USA; 2 Department of Psychiatry, Virginia Commonwealth University, Richmond, VA USA; 3 Department of Child and Adolescent Psychiatry, University Medicine of Gutenberg University, Mainz, Germany; 4 Clinical Research, H. Lundbeck A/S, Copenhagen, Denmark; 5 Biostatistics, H. Lundbeck A/S, Copenhagen, Denmark

**Keywords:** Major depressive disorder, Pediatric patients, Vortioxetine, Long-term use, Safety

## Abstract

Children and adolescents with severe or relapsing major depressive disorder (MDD) may require long-term antidepressant use, but safety and tolerability data on long-term treatment are limited. In a randomized, placebo-controlled trial in children and another in adolescents, vortioxetine and placebo groups showed improvement in MDD symptoms without statistically significant differences between groups. To gain insights on long-term safety and tolerability of vortioxetine in pediatric patients, participants from these two studies were enrolled in two long-term extension studies: 6 months (NCT02871297) followed by another 18 months (NCT03108625). Key safety measures included adverse events (AEs) and Columbia-Suicide Severity Rating Scale (C-SSRS); effectiveness measures included depression symptom severity, cognitive function, and overall functioning. Among the 662 patients in the 6-month extension, 61% experienced a treatment-emergent AE (TEAE), with the most common being nausea (20.8%); 2.1% had a serious AE (SAE), and 6% withdrew because of TEAEs. In the following 18-month extension (*n* = 94), 51% of patients experienced a TEAE, with the most common being headache (13.8%); no SAEs were reported. Based on the C-SSRS, 94% and 96% of patients reported no suicidal ideation or behavior in the 6- and 18-month studies, respectively. During the extension studies, patients continued to show improvement in depressive symptoms and cognitive and overall functioning, with > 50% of patients in remission at the end of each study, regardless of study treatment in the lead-in trial. Overall, vortioxetine remained well tolerated in pediatric patients with MDD who continued in the long-term extension studies with no observed increased risk in suicidal ideation.

**Supplementary Information:**

The online version contains supplementary material available at 10.1007/s00787-024-02560-1.

## Trial registration

ClinicalTrials.gov ID: NCT02871297, August 18, 2016; ClinicalTrials.gov ID: NCT03108625, April 11, 2017.

**Supplementary Information:**

The online version contains supplementary material available at 10.1007/s00787-024-02560-1.

## Introduction

Major depressive disorder (MDD) is a common and chronic psychiatric disorder that is often unrecognized and undertreated in children and adolescents [1]. According to data from the National Survey of Children’s Health, within 2016–2019, an estimated 4.4% of children and adolescents aged 3–17 years had a diagnosis of depression [2]. Reports on current depression during the same time period indicate around 0.1-1.9% of children between the ages of 3-11 years and 5.8% of adolescents between 12-17 years had a current episode [2]. Pediatric presentations of MDD have a diverse profile of symptoms, including variations in appetite, sleep, somatic complaints, irritability, and poor self-esteem, as well as loss of interest in school and activities [1, 3, 4]. Moreover, nearly 50% of pediatric patients with depression remain undiagnosed in the primary care setting [5]. If untreated, children and adolescents with MDD are at higher risk of other mental health disorders, substance use, poor academic and work performance, relational difficulties, and suicidal behaviors, adding to the disease burden and leading to social and functional impairment [1, 3, 6]. In addition, an episode of depression in adolescents can often herald a chronic or relapsing disorder within 5 years [3, 7].

One challenge of pediatric psychiatric care is that a majority of youth with MDD do not seek treatment, or those who do seek help may not receive appropriate care [1, 8]. Currently, only two antidepressants, escitalopram and fluoxetine, are approved by the United States Food and Drug Administration (FDA) for the treatment of MDD in children [1, 9, 10]. Fluoxetine is approved for the treatment of children and adolescents with MDD (aged 8 years and older) in the United States and Europe, and escitalopram is approved for the treatment of adolescents with MDD (aged 12 years and older) in the United States [10]. Several antidepressants with different mechanisms of action have been studied in the pediatric population, including duloxetine, imipramine, desipramine, nortriptyline, sertraline, desvenlafaxine, and vilazodone; however, only negligible reductions in depressive symptoms were observed for most of these treatments compared with placebo and psychotherapy [11, 12]. Among newer antidepressants, only agomelatine has demonstrated efficacy in one study in adolescents [13]. For severe and relapsing MDD, patients may receive long-term antidepressant therapy [14]. Fluoxetine and escitalopram have been studied for long-term use of up to 36 weeks in youth, with no major safety concerns and some symptomatic benefits. However, given the burden of pediatric MDD, there is a need to evaluate additional pharmacological treatments with different mechanisms of action, including their long-term effects associated with use in children and adolescents [6, 15–20].

Vortioxetine is a multimodal antidepressant that acts as an inhibitor of the serotonin transporter and a modulator of various subtypes of serotonin receptors. Vortioxetine is approved by regulatory authorities in more than 80 countries for the treatment of MDD in adult patients at a therapeutic dose range of 5 to 20 mg/day [21, 22]. Vortioxetine has established efficacy and is well tolerated in adults with MDD as short-term therapy and long-term maintenance treatment [22–24]. The safety profile of vortioxetine is consistent in children, adolescents, and adults, with most adverse events (AEs) being mild in severity [21, 25, 26]. Two placebo-controlled, fluoxetine-referenced, short-term trials in children (NCT02709655) and adolescents (NCT02709746) were conducted to evaluate the efficacy and safety of vortioxetine (10 and 20 mg/day) for the acute treatment of MDD [21, 26]. The results of these studies demonstrated a reduction in the Children’s Depression Rating Scale–Revised (CDRS-R) total scores in all treatment groups, with no significant differences between those treated with vortioxetine and placebo [21, 26]. Vortioxetine was well tolerated in both children and adolescents with no major safety concerns [21, 26]. The patients from these studies were enrolled into two long-term extension studies (6 months [NCT02871297] followed by 18 months [NCT03108625]), reported herein. The objective of these studies was to evaluate the safety, tolerability, and effectiveness of long-term use of vortioxetine for up to 2 years in children and adolescents with MDD.

## Methods

### Study design

**Fig. 1:**
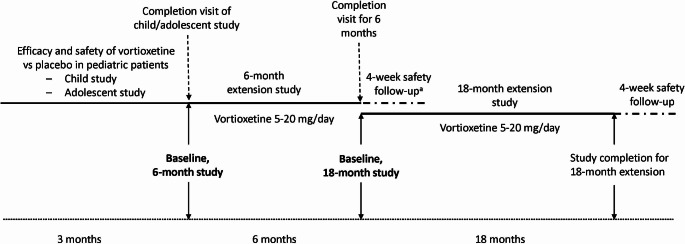
Study design. ^a^A 4-week safety follow-up for patients who did not participate in the 18-month extension study was available

These open-label, long-term extension studies were conducted from August 2016 through April 2022. After completion of the two 12-week (4-week placebo lead-in followed by 8-week vortioxetine treatment) lead-in child and adolescent studies, participants were eligible to enroll in the 6-month long-term extension study, conducted at 78 sites in 20 countries. Participants who completed the 6-month extension were then eligible to enroll in an additional 18-month extension study, carried out at 31 sites in 13 countries. The baseline for the 6-month extension treatment study was the completion visit of the lead-in child or adolescent studies. The baseline for the 18-month extension study was the completion visit of the preceding 6-month extension study **(**Fig. 1**)**. During the 6-month extension, all patients were initiated with 5 mg/day of vortioxetine for the first two days prior to receiving the target dose of 10 mg/day; subsequent dose adjustments to 5, 10, 15, or 20 mg/day were made based on the patient’s response and investigator’s judgment, provided that the patient received the same dose for 2 days before being up-titrated to a new dose. Patients who were then enrolled into the 18-month extension continued on the same dose received at the end of the 6-month extension, with the possibility of dose adjustment to 5, 10, 15, or 20 mg/day based on the patient’s response and investigator’s judgment. The 6-month and 18-month extension studies are registered at ClinicalTrials.gov (NCT02871297 and NCT03108625, respectively).

### Study participants

Male and female patients with a primary diagnosis of MDD according to *Diagnostic and Statistical Manual of Mental Disorders*, *Fifth Edition*, criteria, who completed either the short-term, double-blind child study or the adolescent study were enrolled in the 6-month extension study [21, 26]. Patients who completed the 6-month extension treatment period were eligible to participate in the subsequent 18-month extension study. Detailed eligibility criteria have been described in the preceding short-term, double-blind child and adolescent studies [21, 26]. In brief, key exclusion criteria included comorbid psychiatric conditions such as bipolar disorder, mania, or schizophrenia, clinically relevant neurological, gastrointestinal, cardiovascular, or metabolic disorders, and a suicide attempt within the past 12 months or significant risk of suicide (either in the opinion of the investigators or by answering “yes” to suicidal ideation or suicidal behavior on the Columbia-Suicide Severity Rating Scale [C-SSRS]). Patients taking certain disallowed medications such as some antidepressants, antipsychotics, anticonvulsants, or non-stimulant attention-deficit/hyperactivity disorder (ADHD) medications were restricted from using that medication or were excluded if they recently took the medication or were likely to require the medication during the study. Patients taking a stable dose of stimulant ADHD medication for at least 4 weeks before start of study were allowed to participate. Patients of childbearing potential and sexual maturity followed adequate contraceptive measures. Patients who turned age 18 years during the 6-month extension study were allowed to continue but were not enrolled in the 18-month extension study.

### Study assessments

All safety and efficacy assessments for the 6- and 18-month extension studies were similar and are listed below. Safety assessments included AEs according to the Medical Dictionary for Regulatory Activities (MedDRA) version 22.0, physical and neurological examinations, and changes in laboratory tests, vital signs, height, weight, body mass index (BMI), and electrocardiogram (ECG) parameters from the respective baselines to 6 months and 18 months, which were collected and recorded as AEs if considered clinically significant by the investigators. Tolerability was also assessed using the Pediatric Adverse Event Rating Scale (PAERS) [27], and suicidal ideation and behavior were assessed using the C-SSRS [28] at each study visit. PAERS and C-SSRS are both clinician-rated scales. The PAERS consists of 45 items to evaluate AEs in pediatric patients taking psychotropic medication, and the C-SSRS has four questions addressing suicidal ideation, behavior, and severity [27, 28].

Changes in serum levels of reproductive hormones such as estradiol, testosterone, luteinizing hormone, follicle-stimulating hormone, luteinizing hormone-releasing hormone, and prolactin were assessed. Additionally, Tanner scores and length of menstrual cycle were evaluated to examine sexual maturity and functioning.

Depressive symptom assessments included change from the respective baselines to 6 months and 18 months in the CDRS-R total score; Clinical Global Impression–Severity (CGI-S) and CGI-Improvement (CGI-I) were assessed relative to baseline or enrollment in the lead-in placebo-controlled studies. The CDRS-R total score was used to measure the severity of depressive symptoms among the study participants, and consists of 17 items, with scores ranging from 17 (normal) to 113 (severe depression) [29]. The CGI-S, a 7-point scale ranging from 1 (normal–not at all ill) to 7 (among the most extremely ill patients), was used to measure the severity of illness [30]. The CGI-I, a 7-point scale ranging from 1 (very much improved) to 7 (very much worse), was used to measure the clinician’s impression of the patient’s improvement (or worsening) in reference to baseline or enrollment in the lead-in placebo-controlled studies [30]. Remission was defined as CDRS-R total score ≤ 28 (minimal or no symptoms), or a CGI-S score of ≤ 2, response as a CGI-I score ≤ 2, and relapse as CDRS-R total score ≥ 40 during the treatment period.

Cognitive function was assessed using change from the respective baselines to 6 months and 18 months in the Behavior Rating Inventory of Executive Function–Preschool (BRIEF-P) and BRIEF–Self-Report (BRIEF-SR) scores. The BRIEF-P and BRIEF-SR scores were used in patients aged 7–11 years and 11–18 years, respectively, to measure their executive function behavior in an everyday environment. Both of these versions contain 86 items and are rated on a 3-point scale from N (never) to O (often) [31], where a higher score may indicate difficulty with some aspects of executive functioning.

Functionality was assessed using change from the respective baselines to 6 months and 18 months in Children’s Global Assessment Scale (CGAS) and Pediatric Quality of Life Inventory Present Functioning Visual Analogue Scales (PedsQL™ VAS) total scores. The CGAS ranges from 1 (most functionally impaired child) to 100 (healthiest), with a score greater than 70, indicating normal functioning [32]. The PedsQL™ VAS was used to assess at-that-moment functioning in patients by assessing total score (mean of six items: anxiety, sadness, anger, worry, fatigue, and pain) and Emotional Distress Summary Score (mean of four items: anxiety, sadness, anger, and worry) [33]. Adherence was assessed by vortioxetine plasma concentration at 6 months and by tablet count for both the 6- and 18-month extension studies. A detailed list of assessment timelines is available in the Supplementary Table.

### Statistical analysis

Both the 6-month and 18-month studies followed the same statistical methods described below. Safety assessments were performed using the all-patients-treated set (APTS) of each study, which included patients who received at least one dose of vortioxetine. The efficacy assessments were performed using the full analysis set (FAS) of each study, which included all patients with a valid respective baseline visit and at least one post-baseline CDRS-R total score during the respective study period. AEs, clinical safety laboratory test values, vital signs, body measurements (height, weight, BMI), ECG parameters, pubertal stages, menstrual cycle, and C-SSRS scores were summarized in each study using descriptive statistics. All effectiveness assessments as well as pharmacokinetic (PK) and adherence data for each study were summarized using descriptive statistics. Statistical Analysis System version 9.4 was used for all analyses.

## Results

### Patient disposition

**Fig. 2:**
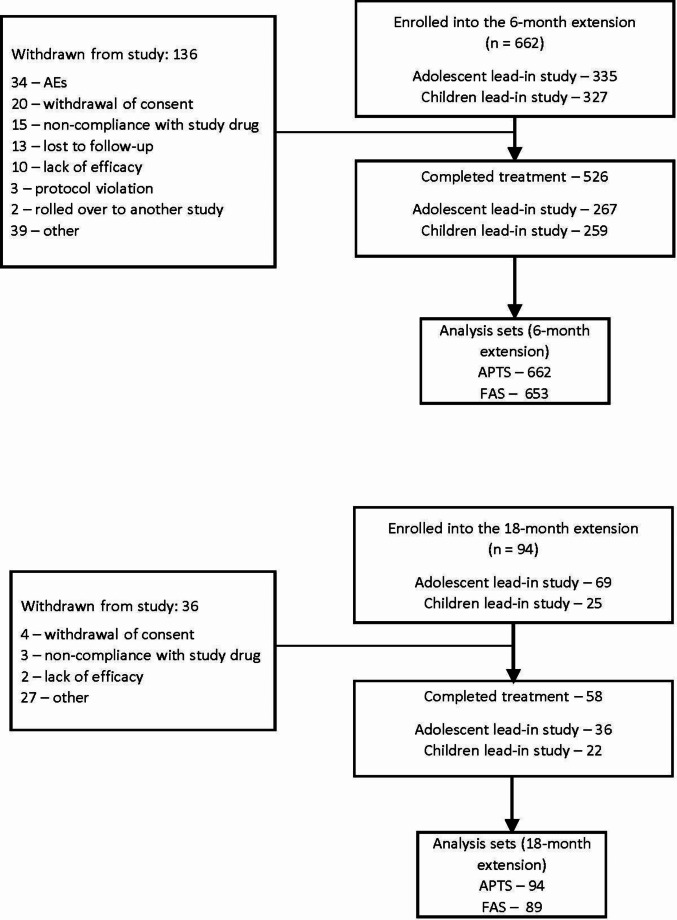
Patient disposition. *AEs* = adverse events; *APTS* = all-patients-treated set; *FAS* = full analysis set; *n* = number of patients

In the 6-month extension study, 662 patients were enrolled (APTS), of whom 79.5% (*n* = 526) completed the study, and 98.6% (*n* = 653) were included in the FAS for efficacy assessments (Fig. 2). Overall, 20.5% (*n* = 136) of the patients withdrew from the 6-month study. The most common primary reasons for withdrawal (> 4%) were “other” (5.9%, *n* = 39) and “adverse event” (5.1%, *n* = 34); six patients in the “other” category were withdrawn due to study termination based on the negative outcome of the short-term, double-blind, lead-in studies. In the 18-month extension study, 94 patients who completed the 6-month extension study were enrolled after which enrollment was stopped as requirement for sample size was met. All 94 were treated (APTS), of whom 61.7% (*n* = 58) completed treatment, and 94.6% (*n* = 89) were included in the FAS for efficacy assessments. A total of 38.3% (*n* = 36) of patients withdrew from the study. The most common primary reasons for withdrawal (> 4%) were “other” (28.7%, *n* = 27) and “withdrawal of consent” (4.3%, *n* = 4). Reasons for withdrawal marked as “other” included sponsor’s decision to withdraw adolescent patients based on the negative outcome of the lead-in adolescent study (*n* = 10), withdrawal of consent (*n* = 4), refusal to attend (*n* = 4), relocation or logistics (*n* = 3), not requiring medication anymore (*n* = 3), and 1 patient each due to non-adherence (parental concern), disallowed concomitant medication, or were in remission for a year. No patients withdrew due to an AE during the 18-month extension study.

### Demographic and baseline characteristics

The demographic characteristics of the patients are provided in Table 1. In the 6-month extension study, 45.3% of patients were children (7–11 years) and 54.7% were adolescents (12–17), whereas in the 18-month extension study, 22.3% were children and 77.7% were adolescents. Approximately half the children were prepubertal (Tanner stage I), while among adolescents, a majority were in pubertal stages (Tanner stages II to IV) at the 6- and 18-month baselines. At the 6-month study baseline, the mean CDRS-R total score was 44.45, which further decreased to 33.42 at the 18-month study baseline.

**Table 1:** Patient demographics

Characteristics	6-month extension study	18-month extension study
**Number of patients**	**662**	**94**
Age, mean (SD)	12.33 (3.07)	13.88 (2.62)
Age groups, n (%)		
Children (7–11)	300 (45.3)	21 (22.3)
Adolescents (12–18)	362 (54.7)	73 (77.7)
Sex, n (%)		
Male	301 (45.5)	39 (41.5)
Female	361 (54.5)	55 (58.5)

### Safety and tolerability outcomes

#### Adverse events

In the 6-month extension study, 61% (*n* = 404) of patients reported treatment-emergent AEs (TEAEs), with the majority being mild to moderate, and 3.9% (*n* = 26) of patients experiencing a severe TEAE. As per the treatment, during the lead-in, double-blind, child and adolescent studies, the incidence of TEAEs in the 6-month extension study was similar among patients who received placebo (61.5%), vortioxetine 10 mg/day (58.0%), vortioxetine 20 mg/day (64.1%), and fluoxetine 20 mg/day (60.0%); nausea was reported in 26.0%, 19.5%, 19.6%, and 17.8%, for patients who received placebo, vortioxetine 10 mg/day, vortioxetine 20 mg/day, and fluoxetine 20 mg/day, respectively, in the lead-in studies. Overall, approximately 37% (*n* = 242) of patients experienced TEAEs that were considered causally related to the treatment. A total of 6.0% (*n* = 40) of patients withdrew because of a TEAE in the 6-month extension study, with nausea (1.7%; *n* = 11) being the most common TEAE leading to withdrawal, followed by suicidal ideation and suicide attempt (*n* = 4; 0.6% each). A total of 2.1% (*n* = 14) of patients reported serious AEs (SAEs), of which events related to psychiatric disorders were most common and were observed in 1.4% (*n* = 9). The SAEs that were reported in > 1 patient in the treatment period were suicide attempt (4 patients, 0.6%), suicidal ideation (4 patients, 0.6%), and intentional overdose (3 patients, 0.5%). TEAEs with an incidence of ≥ 5% in the 6-month study included nausea, headache, vomiting, nasopharyngitis, and abdominal pain (Table 2).

During the 18-month extension study, 51% (*n* = 48) of patients reported TEAEs, the majority of which were mild to moderate, with only 1 patient experiencing a severe TEAE. Approximately 18% (*n* = 17) of patients experienced TEAEs that were considered causally related to treatment. In the 18-month extension study, no patients withdrew because of a TEAE, and no SAEs or fatal events were reported. TEAEs with an incidence ≥ 5% in the 18-month extension study included nausea, headache, vomiting, nasopharyngitis, and abdominal pain (Table 2).

**Table 2:** TEAEs with ≥ 5% incidence

Preferred terms	6-month extension study	18-month extension study
**Total number of patients**	**662**	**94**
Patients with TEAEs, n (%)	255 (38.5)	30 (31.9)
**Incidence of TEAEs**,** n (%)**		
Nausea	138 (20.8)	7 (7.4)
Headache	116 (17.5)	13 (13.8)
Vomiting	69 (10.4)	5 (5.3)
Abdominal/upper abdominal pain	46 (6.9)	5 (5.3)
Nasopharyngitis	46 (6.9)	6 (6.4)
Dizziness	38 (5.7)	0
Hyperprolactinemia	0	5 (5.3)
Respiratory tract infection (viral)	0	5 (5.3)

*TEAEs* = treatment-emergent adverse events

There was a general decrease over time in the incidence and intensity of the signs and symptoms collected using the PAERS in both studies. The most common symptoms appeared to be related to MDD (mood-related symptoms, anxiety) and the comorbid disorder of ADHD (hyperactivity, attention difficulties). The symptoms also reflected some of the AEs in these studies, such as somnolence, stomachache, and nausea. In both studies, the majority of the PAERS symptoms were either mild or moderate.

### Suicide-related events

Results from the C-SSRS assessment in the 6-month extension study showed that 94% (617/659) of patients had no suicidal ideation or behavior during the study; five patients (0.8%) had suicidal behavior reported as SAEs (nonfatal suicide attempt, *n* = 4; suicidal behavior, *n* = 1), five patients (0.8%) had nonspecific active suicidal thoughts, and five patients (0.8%) had active suicidal ideation without intent to act. Suicidal ideation and suicide attempt were each reported by four patients (0.6%) as TEAEs leading to withdrawal from the 6-month extension period.

As per the C-SSRS assessment in the 18-month extension study, 96% (90/94) of patients had no suicidal ideation or behavior; 4.3% (*n* = 4) had suicidal ideation without intent to act, and no patients had suicidal behavior. One patient had a suicide-related TEAE captured by standardized MedDRA suicide/self-injury queries that was considered mild and not related to treatment.

### Laboratory and vital signs findings

In the 6-month extension study, mean changes from baseline to 6 months were small, and no consistent trends were observed for clinical safety laboratory tests, height, weight, BMI, vital signs, or ECG. Similarly, in the 18-month extension study, mean changes from baseline to 18 months were small, and no consistent trends were observed for clinical safety laboratory parameters, vital signs, height, weight, or BMI, except for prolactin, which showed an increase in mean score in five patients. At the end of the treatment period, these levels decreased to the baseline values for three patients, and the other two patients were not tested after the end of study. In addition, one patient showed a shift from normal weight to obese during the 18-month extension study. In both the 6- and 18-month studies, there were no notable changes observed in reproductive hormones, Tanner score, or menstrual cycle duration from respective baselines to either the 6-month or 18-month time points.

### Effectiveness outcomes

In the 6-month extension study, the change in mean CDRS-R total score and mean CGI-S from baseline to 6 months was − 16.7 and − 1.5 points, respectively, showing improvement in MDD symptom severity (Supplementary Table). Likewise, the CGI-I score was 1.7 points at 6 months, suggesting improvement. At 6 months, more than 85% of patients demonstrated a response (CGI-I score ≤ 2) and 59% were in remission (CDRS-R total score ≤ 28) (Table 3). Remission rates were similar for patients from both short-term, double-blind, lead-in studies, with 58% of patients from the child study and 60% from the adolescent study in remission at 6 months. Cognitive improvement was demonstrated through reduction in BRIEF-P and BRIEF-SR scores from baseline to 6 months by > 7 points. The CGAS scores increased by more than 14 points from baseline to 6 months, indicating improvement in overall functioning. Confirming these assessments, patients also reported improvement in PedsQL™ VAS total and emotional distress scores.

In the 18-month extension study, the change in mean CDRS-R total score and mean CGI-S from the baseline to 18 months was − 8.9 and − 1.3 points, respectively, showing further improvement in MDD symptoms from the end of the 6-month study (Table 3). The CGI-I score was 1.4 points at 18 months, also showing improvement. A total of 84% of patients were in remission (CDRS-R total score ≤ 28) at 18 months (Table 3); 87% of patients from the double-blind child lead-in study and 82% from the adolescent study were in remission at 18 months. At 18 months, the reduction in BRIEF-P scores was > 7 points and the reduction in BRIEF-SR score was > 11 points from baseline. The CGAS scores increased by more than 11 points from baseline to 18 months, indicating good overall functioning, and similar improvements were noted through the PedsQL™ VAS total and emotional distress scores.

**Table 3:** Response and remission analysis

	6-month extension study	18-month extension study
CDRS-R remission, n (%)	300 (59.3)	57 (83.8)
CGI-S remission, n (%)	348 (68.8)	63 (96.9)
CGI-I response, n (%)	436 (86.2)	–
CDRS-R relapse, n (%)	77 (15.2)	0

*CDRS-R* = Children’s Depression Rating Scale–Revised; *CGI-I* = Clinical Global Impression–Improvement; *CGI-S* = CGI-Severity; *–* = not evaluated in the 18-month extension study

### Treatment course and adherence

During the 6-month extension study, the mean total duration of exposure based on tablet count was approximately 160 days (~ 5 months) and was similar in patients from both the child and adolescent double-blind, lead-in studies. The mean dose of vortioxetine was 13 mg/day, with patients from the child lead-in study generally receiving a slightly lower mean dose than those from the adolescent study (12 mg/day vs. 13 mg/day). Most patients (≥ 97%) demonstrated ≥ 80% adherence to the treatment based on the tablet count; however, 110 (25%) of the 434 patients treated with vortioxetine demonstrated nonadherence based on plasma concentrations in terms of estimated apparent total plasma clearance of drug values (estimated CL/F > 120 L/h) and PK samples below the first lower limit of quantification at any visit when it was assessed. During the 18-month extension study, approximately 74% of patients received vortioxetine for at least 15 months; a majority of patients (98.9%) received the intended dose of vortioxetine and demonstrated ≥ 80% adherence to treatment based on tablet count. The mean dose of vortioxetine was 13 mg/day, with patients from the child lead-in study generally receiving a lower mean dose than those from the adolescent study (11 mg/day vs. 14 mg/day). The modal dose of vortioxetine was 10 mg for both the 6- and 18-month extension studies, with similar mean doses of 12.4 mg at 6 months and 13.2 mg at 18 months (Table 4).

**Table 4:** Exposure to vortioxetine at 6 and 18 months

	6-month extension study	18-month extension study
All patients		
Mean (SD)	12.4 mg (4.5)	13.2 mg (5.0)
Modal dose^a^	10 mg	10 mg
Mean (SD) at study completion/withdrawal	12.6 mg (5.0)	12.7 mg (5.4)
**Patients from the child lead-in study**		
Mean (SD)	11.6 mg (4.4)	11.3 mg (4.7)
Modal dose^a^	10 mg	10 mg
**Patients from the adolescent lead-in study**		
Mean (SD)	13.2 mg (4.4)	13.88 mg (4.9)
Modal dose^a^	10 mg	15 mg

^a^Median of modal dose received by each patient

## Discussion

In these studies, flexible dosing of vortioxetine from 5 to 20 mg/day was generally safe and well tolerated in pediatric patients with MDD who continued treatment for up to two years after completing a placebo-controlled efficacy study [21, 26]. The safety and tolerability profile of vortioxetine in pediatric patients after long-term use was comparable to what has been observed in pediatric patients after short-term use. No new important risks were identified in the pediatric population beyond those established for the adult population. Although the short-term, placebo-controlled studies evaluating the efficacy and safety of vortioxetine in children and adolescents with MDD did not meet their primary endpoint, patients in all treatment groups had similar improvements in depressive symptoms over the course of the studies. During the long-term extension studies, vortioxetine treatment was associated with continued improvements in symptoms of MDD, although these conclusions are limited by lack of a comparator.

The choice of vortioxetine dose (5, 10, 15, or 20 mg/day) in the current study was based on knowledge from the clinical studies in adult patients and from the pediatric vortioxetine PK and tolerability study, which was designed to provide supportive information for appropriate dosing regimens for clinical trials in pediatric populations [22–24]. The results of the overall studies in adults and the pediatric PK study demonstrated that the exposure, safety, and tolerability of vortioxetine in children are comparable to those in adults, thus supporting use of vortioxetine 5 to 20 mg/day for clinical studies of vortioxetine in pediatric patients [22, 23, 25]. Most patients in the long-term extension studies remained on a daily dose of 10 to 15 mg of vortioxetine. In the 6-month extension study, the percentages of patients who received the highest and lowest dose levels were 25% and 22.8%, respectively, while in the 18-month extension study the percentages of patients who received the highest and lowest dose levels were 31.9% and 21.3%, respectively.

Vortioxetine was found to be well tolerated in long-term use, with a safety profile similar to that in adults [24]. During the 6- and 18-month extension studies, the incidence of AEs was relatively low, and most TEAEs were considered to be either mild or moderate. While the pathophysiological reasoning for these AEs is not entirely clear, there exists a relationship to serotonergic transmission modulation, which has some mechanistic considerations on sleep and sexual AEs [34]. Furthermore, the safety and tolerability profile of vortioxetine after long-term use in these studies was similar to that observed in pediatric patients after short-term use [21, 26], and no new important risks were detected beyond those established for the adult population [19, 22]. Some studies indicate that selective serotonin reuptake inhibitors tend to elicit a higher incidence of AEs and reactions that occur in the first few weeks of use compared with longer-term usage [35]. Patients who received placebo in the lead-in studies and switched to vortioxetine in the long-term extension studies had an overall incidence of AEs (61%) similar to patients who continued on vortioxetine treatment (58-64%); the incidence of nausea was slightly higher in patients switching to vortioxetine from placebo (26.0%) compared with those continuing vortioxetine (19.5-19.6%). Additionally, these studies reported no new safety concerns based on clinical laboratory, ECG, or vital sign measurement results or changes in Tanner score.

Treatment-related suicidal ideation and behavior are areas of concern in pediatric patients with MDD [6, 36]. In this study, suicidality was monitored using the C-SSRS assessment, and results suggested that suicidal ideation or risk of suicide did not increase in patients receiving vortioxetine for long-term use. Similarly, Findling et al. reported suicidal ideation in only one pediatric patient with long-term use of vortioxetine in a 6-month open-label study [16]. A comparatively higher rate of suicidal ideation was reported in other long-term treatment or extended studies using duloxetine (4-10% of patients) and in patients treated with escitalopram compared with placebo [6, 15, 18].

Efficacy of vortioxetine for the treatment of children and adolescents with MDD was not demonstrated in either of the two placebo-controlled, lead-in studies; similar symptom improvements were observed across all treatment groups. Changes in CDRS-R total, CGI-S, and CGI-I scores suggested a reduction in MDD symptom severity with use of vortioxetine during the 6- and 18-month extension studies in both children and adolescents who opted to continue, with a majority of patients in remission toward the end of the study. Additionally, CGAS as well as BRIEF-P and -SR scores indicated improvements in overall and executive functioning, respectively. In the previous long-term study of vortioxetine by Findling et al., improvements that occurred in the lead-in period were maintained throughout the 6-month open-label period, as observed in this study [19].

### Limitations

Due to the open-label nature of these studies and given that vortioxetine did not show a difference compared with placebo in either of the lead-in studies, the efficacy of vortioxetine in children and adolescents with MDD should be interpreted with caution. Although continued improvements were observed in depressive symptoms and other parameters such as cognitive and overall functioning over the course of the study, they may have resulted from therapeutic effects of the study procedures themselves, been a reflection of the natural course of the disease, or been a result of practice effects. In addition, participants in these studies were enrolled based on the stringent eligibility criteria for the double-blind studies, which limits generalizability of these results to the general clinical population, who may have comorbid psychiatric disorders.

### Conclusions

These 6-month and 18-month open-label extension studies provide evidence that treatment with vortioxetine (5 to 20 mg/day) was generally safe and well tolerated in youth with MDD who continued treatment up to two years. The safety and tolerability profile of vortioxetine in children and adolescents after long-term use was comparable to what has been observed in pediatric patients after short-term use. No new risks were identified in the pediatric population beyond those established for the adult population. Improvements in depressive symptoms (as assessed using the CDRS-R and CGI-I scales), cognitive function (as assessed using the BRIEF-P and -SR scales), and overall functioning (as assessed using the CGAS and PedsQL™ VAS) were also observed.

## Electronic supplementary material

Below is the link to the electronic supplementary material.

Supplementary Material 1

## Data Availability

The data supporting the findings of this study are included in the article; further inquiries can be directed to the corresponding author.
