# Supplementary material for: Vortioxetine in children and adolescents with major depressive disorder: 6-month and 18-month open-label, flexible-dose, long-term extension studies
Source: Eur Child Adolesc Psychiatry. 2024 Sep 6;34(4):1425–34. doi: 10.1007/s00787-024-02560-1 (PMC12000202; doi:10.1007/s00787-024-02560-1)
Supplement: Supplementary file 1 — Supplementary Material 1 [file 787_2024_2560_MOESM1_ESM.pdf]

Supplementary material for: Vortioxetine in children and adolescents with major depressive disorder: 6-month and 18-month open-label, flexible-dose, long-term extension studies

DelBello MP, et al.

Supplementary Table. Mean Change in Effectiveness Outcomes

Efficacy outcomes	6-month extension study			18-month extension study		
	**6-month study baseline, mean (SD)**	**6 months, mean (SD)**	**Change from baseline to 6 months, mean (SD)**	**18-month study baseline, mean (SD)**	**18 months, mean (SD)**	**Change from baseline to 18 months, mean (SD)**
CDRS-R	44.5 (14.8) (n=653)	28.8 (10.6) (n=506)	–16.7 (14.7)	33.4 (11.8) (n=89)	23.2 (7.4) (n=68)	–10.6 (9.8)
CGI-S	3.5 (1.1) (n=653)	2.1 (1.1) (n=506)	–1.5 (1.2)	2.6 (1.1) (n=89)	1.3 (0.6) (n=65)	–1.4 (1.1)
BRIEF-P GEC	57.7 (12.0) (n=290)	51.4 (12.7) (n=217)	–7.4 (11.7)	57.1 (12.4) (n=20)	48.1 (12.6) (n=17)	–8.7 (8.7)
BRIEF-P MCI	57.9 (12.7) (n=290)	52.0 (13.0) (n=219)	–7.4 (12.0)	56.8 (12.2) (n=20)	48.7 (12.6) (n=17)	–7.9 (7.9)
BRIEF-SR GEC	57.9 (15.3) (n=351)	50.0 (16.1) (n=272)	–7.5 (13.8)	54.9 (16.0) (n=65)	43.9 (12.5) (n=47)	–11.5 (14.1)
BRIEF-SR MCI	58.3 (15.5) (n=351)	50.8 (15.6) (n=277)	–7.2 (13.7)	55.3 (14.9) (n=65)	44.6 (12.3) (n=47)	–11.2 (13.2)
CGAS	66.0 (14.2) (n=653)	79.6 (12.7) (n=506)	14.8 (14.3)	72.9 (12.8) (n=89)	86.8 (9.4) (n=65)	14.3 10.6)
PedsQL™ VAS total	2.9 (2.1) (n=653)	1.5 (1.6) (n=505)	–1.5 (1.9)	1.9 (1.8) (n=89)	1.1 (1.4) (n=65)	–0.7 (1.4)
PedsQL™ VAS total ED	2.8 (2.2) (n=653)	1.4 (1.7) (n=505)	–1.5 (2.0)	1.7 (1.8) (n=89)	1.1 (1.4) (n=65)	–0.7 (1.5)
CGI-I	2.7 (1.0) (n=653)	1.7 (0.8) (n=506)	–	2.1 (0.9) (n=89)	1.4 (0.6) (n=65)	–

BRIEF-P=Behavior Rating Inventory of Executive Function-Parent; *BRIEF-SR*=BRIEF-Self Reported; *CDRS-R*=Children's Depression Rating Scale–Revised; *CGAS*=Children's Global Assessment Scale; *CGI-I*=Clinical Global Impression–Improvement; *CGI-S*=CGI-Severity; *ED*=emotional distress; *GEC*=Global Executive Composite; *MCI*=Metacognition Index; *PedsQL™ VAS*=Pediatric Quality of Life Inventory Present Functioning Visual Analogue Scale; – =no value found.
